# Single-step aerosol-based synthesis of nanostructured thin films for hydrogen sensing†

**DOI:** 10.1039/d4na00884g

**Published:** 2025-02-13

**Authors:** Klito C. Petallidou, Peter Kováčik, Andreas Schmidt-Ott, George Biskos

**Affiliations:** a Climate and Atmosphere Research Centre, The Cyprus Institute 2121 Nicosia Cyprus g.biskos@cyi.ac.cy; b National Research Council of Canada K1A 0R6 Ottawa Canada; c Faculty of Applied Sciences, Delft University of Technology 2629 HZ Delft The Netherlands a.schmidt-ott@tudelft.nl; d VSPARTICLE B.V 2629 JD Delft The Netherlands; e Faculty of Civil Engineering and Geosciences, Delft University of Technology 2628 CN Delft The Netherlands g.biskos@tudelft.nl

## Abstract

This article describes a single-step method for synthesizing nanostructured materials using evaporation–condensation synthesis and inertial impaction of aerosol nanoparticles. The as-deposited films exhibit anisotropic vertical and horizontal sintering of their palladium nanoparticle building blocks, yielding vertical structures. The electrical conductivity of the films is stable and highly sensitive to the presence of hydrogen in the overlaying gas, at concentrations that range from a few hundreds parts per million to a few percent.

## Abstract 2

Single-step synthesis method for producing sintered nanoparticle-based films for highly sensitive hydrogen sensing.

## Graphical abstract

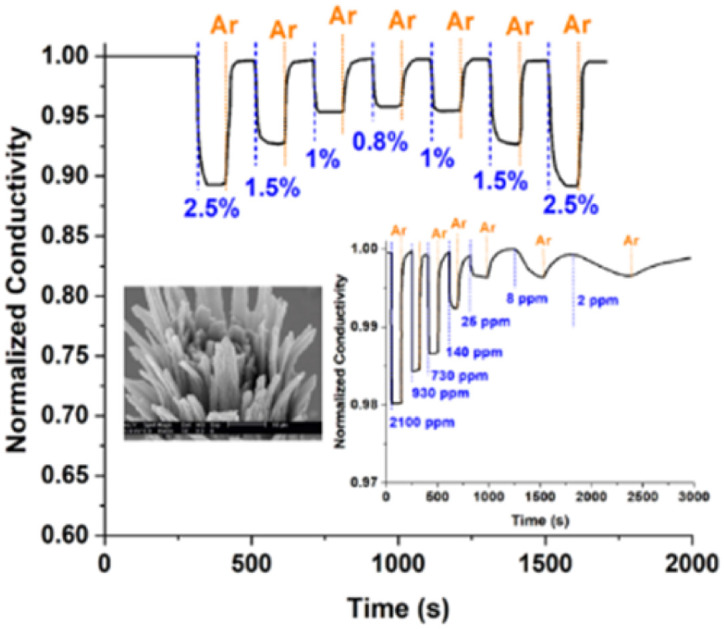

Nanoparticle-based materials offer unique advantages for various applications,^1–3^ as they can be tailored to have specific properties, including high surface-to-volume ratio and enhanced reactivity among others. These advantages can be attributed to their nanoparticle (NP) building blocks, which can be engineered to have specific sizes (from 1 to 100 nm) and composition,^4^ depending on the application. Bottom-up NP synthesis can be achieved by wet chemistry (*e.g.*, sol–gel or precipitation) or gas-phase methods (*e.g.*, flame spray pyrolysis, as well as evaporation–condensation methods employing glowing wires or arc/spark discharges). The latter can be advantageous due to their high versatility with respect to the size and chemical composition of the resulting NPs,^5,6^ while being simple and environmentally friendly as they do not produce any wastes.^3^ Coupled with diffusional, inertial or electrostatic deposition,^7,8^ NP synthesis in the gas phase can offer versatile approaches for assembling NP-based thin films of high porosity.

Gas-phase NP synthesis can also offer promising solutions for the hydrogen-based economy, through a number of novel applications for its production^9,10^ and detection/sensing.^2,11–13^ Hydrogen has a substantial energy content per unit weight while its combustion produces only water as a byproduct, making it a highly attractive option as a fuel.^14^ At the same time, however, it is extremely flammable and explosive at concentrations above 4% in ambient air, posing significant risks when storing and using it. This requires effective and robust tools for detecting and measuring the concentration of hydrogen in the atmospheric environment.^15^ Apart from safety applications, H_2_ detectors and sensors are important to our endeavor in extraterrestrial explorations as the presence of the gas in the atmosphere of other planets provides a strong indication of life.^16^

There are a number of approaches for sensing H_2_ in ambient air.^17^ Among those, electrochemical and chemiresistive sensors provide attractive solutions as they have excellent sensitivity and are not expensive to produce.^2,18,19^ In general, the operating principles of electrochemical and/or chemiresistive hydrogen sensors rely on the interaction of H_2_ with the sensing material, which in turn alters its electrical properties. Electrochemical H_2_ sensors probe changes in electrical current through electrochemical cells induced by redox reactions of H_2_ on the surface of electrodes,^18^ while chemiresistive H_2_ sensors rely on changes in electrical resistance/conductance resulting from the interaction between H_2_ and the sensing material.^2^ Among the two, chemiresistive sensors are more simple and thus less expensive to produce,^20,21^ but there is still room for improving their performance; *e.g.*, making them faster to respond, more sensitive and more selective.^2^

Pd is one of the most effective elements for H_2_ sensing,^21–23^ as it easily reacts with hydrogen, even at room temperature, to form palladium hydride that has a higher resistivity compared to pure Pd.^24,25^ The resistance of Pd-based materials typically increases non-linearly with increasing hydrogen concentration, because PdH_*x*_ goes through three different phases: the α-PdH_*x*_ phase at low H_2_ concentrations ([H_2_] ≤ 1%); a phase where both α and β phases co-exist at intermediate concentration (1% ≤ [H_2_] ≤ 2%); and the β-PdH_*x*_ phase at elevated hydrogen levels ([H_2_] ≥ 2%).^26^ In any case, producing sensors with a low-enough Limit of Detection (LoD) and high sensitivity, requires synthesizing porous sensing materials with a large surface-to-volume ratio.

The NP-based thin films tested in this work were prepared using the experimental set-up illustrated in Fig. 1. The set-up consisted of a glowing wire generator (GWG) of aerosol NPs,^27^ followed by a focusing impactor that creates a narrow NP beam in which the NPs are accelerated and deposited on the substrate. Pd NPs were synthesized using a coiled Pd wire (50 to 100 mm long, and 0.5 mm in diameter; 99.95% purity), fixed in the GWG chamber. The material was heated close to its melting point by applying a current of *ca.* 10 A (we should note here that the evaporation rate of Pd at its melting point, *T*_M_ = 1828 K, is 4.32 × 10^−2^ mol m^−2^ s^−1^).^27^ The resulting vapors were subsequently quenched and carried away by a gas flow (2 L min^−1^ of pure Ar, 99.999% purity), forming NPs upon nucleation and growth.

**Fig. 1:**
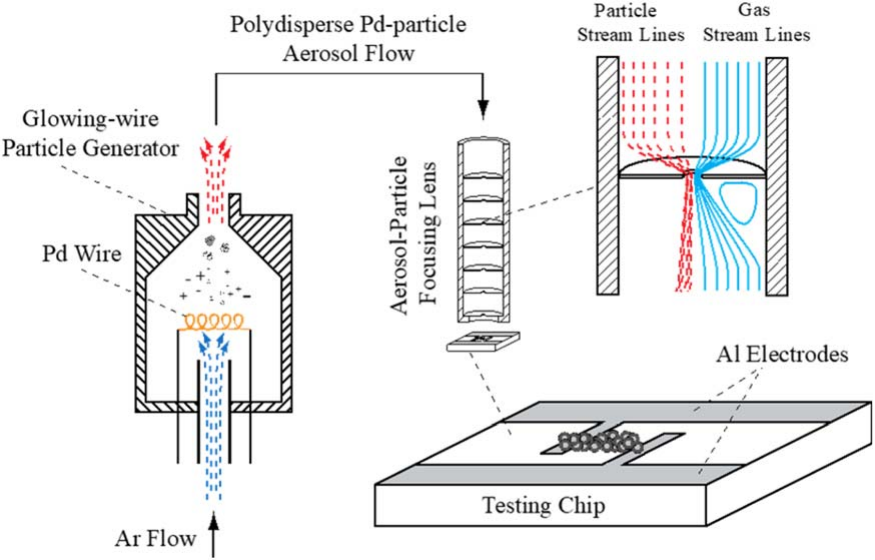
Schematic layout of the experimental set-up developed and build to synthesize the Pd-based films for H_2_ sensing. The system consists of a glowing wire aerosol NP generator, and an aerosol focusing impactor.

The Pd NPs having sizes in the range of 10–30 nm (*cf.* Fig. S1 in the ESI†) were deposited by focusing inertial impaction on the sensing chip. The stream of NPs was focused by a set of aerodynamic lenses having 4 stages, (*i.e.*, 4 orifices with a diameter of 3.8 mm), creating a NP beam with a diameter of less than a couple of hundred microns.^28–31^ The assembly of the focusing lenses was mounted on a traverse that could move in one dimension over the electrode, thereby allowing us to create lines of deposited NPs. A glass substrate (dimensions: 20 × 15 mm) with two aluminum electrodes (*cf.*Fig. 1), was used as a sensing chip. The aluminum electrodes were 1 mm apart from each other and the NPs were deposited as lines over a period of 15 minutes to connect the two electrodes. The length of the deposited lines was 3 mm, whereas the broadness and height of the deposited lines ranged respectively within 120–150 μm and 200–300 nm.

Fig. 2 shows Scanning Electron Microscopy (SEM; JEOL JSM-6010LA) images of the produced NP-based thin films. Evidently, the nanostructured films are uniform along the entire length of the deposition (*cf.*Fig. 2a, b and e). Fig. 2c and d show details of the starting and ending point where the deposition was prolonged. More specifically, Fig. 2c shows the morphology of the structure in its intact state, whereas Fig. 2d the deposition after the tip was deliberately broken to observe the internal structure of the material. As shown in these images, the selected NP deposition process (*i.e.*, focused inertial impaction; *cf.*Fig. 1) creates vertical structures (*i.e.*, along the direction of the deposition) formed by the complete sintering of the deposited NPs. This is not surprising considering that the aerosol NPs produced by the GWG are accelerated roughly to sonic speed in the focusing impactor, picking up kinetic energy that is converted to heat upon collision with the NPs that have already been deposited. As a result, the NPs melt around the points of contact upon collision, forming vertical structures. Horizontal sintering also occurs because the vertical structures are in close proximity, allowing neighboring NPs to melt at their contact points. This lateral interaction between adjacent NPs facilitates the formation of horizontally sintered connections, complementing the vertical sintering driven by the inertial deposition process. The vertical structures appear bent (as shown in Fig. 2d) due to mechanical stresses keeping them together.

**Fig. 2:**
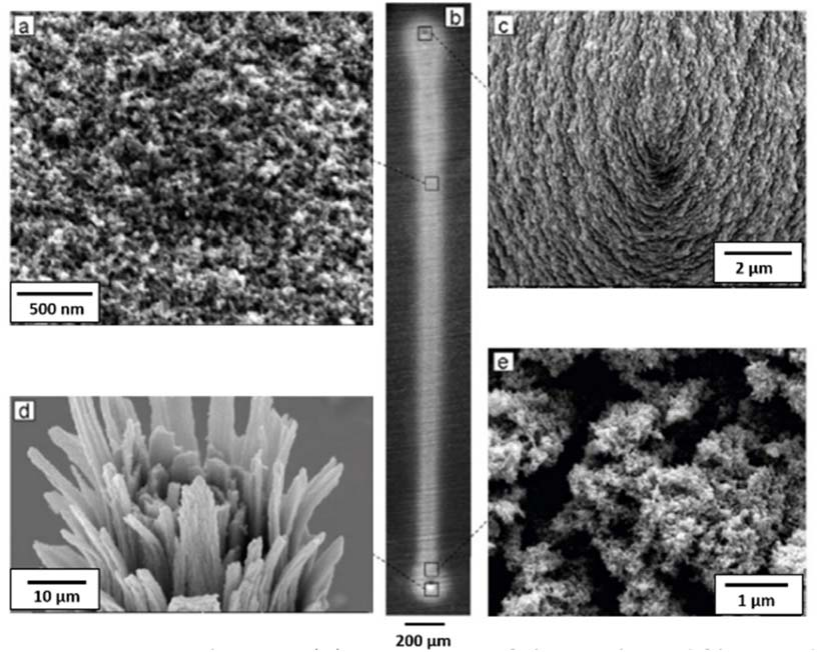
SEM images showing (a) a top view of the NP-based film produced by the set-up illustrated in Fig. 1, (b) a line print of the NP-based film on a metallic substrate, (c) top view of the cone at the one end of the printed line formed by an extended NP deposition time, (d) top view of the cone at the other end of the printed line after the tip was broken off, and (e) top view of the NP-based film at the end cone.

We evaluated the sensing properties of the resulting material following the method described in Isaac *et al.*,^7^ using mixtures of H_2_ in Ar at different concentrations. Fig. 3 shows changes in the conductivity of the Pd NP-based film for H_2_ concentrations cycled from 0.8 to 2.5%. These measurements show that the sensing material exhibits very good reproducibility, with respective responses (determined as (1 − *σ*_H_2_/Ar_/*σ*_Ar_) × 100, where *σ*_Ar_ is the conductivity of the material when exposed to pure Ar and *σ*_H_2_/Ar_ to Ar containing specific concentrations of H_2_) of 11.0 and 4.7%, and response times (*i.e.*, the time needed to reach 90% of the maximum signal after exposing it to H_2_) of 21.3 and 7.7 s when exposed to 2.5 and 1.0% H_2_, respectively. The inset in Fig. 3 shows changes in the conductivity of the films when the H_2_ concentration of the overlaying gas was progressively reduced from *ca.* 0.2% to 2 ppm. The response and response time at the lowest H_2_ concentration we tested here (*i.e.*, 2 ppm) were respectively 0.3% and 400 s. We should also note here that our results demonstrate reproducibility of ±10% across different sensors, confirming the reliability of the manufacturing and measurement process (results not shown).

**Fig. 3:**
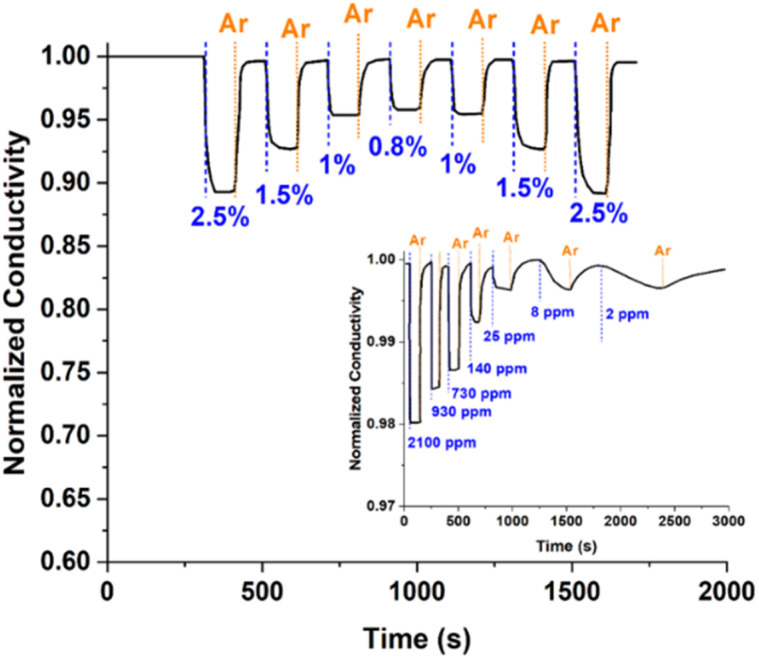
Changes in the conductivity of the Pd NP-based films upon cycling the concentration of H_2_ in the overlaying gas from 0.8 to 2.5%. Inset: Changes in the conductivity of the films when the concentration of H_2_ was cycle-reduced from 2100 to 2 ppm. The dashed blue lines indicate the times when the films were exploded to H_2_, while the dotted orange lines mark the introduction of Ar in the sensing set-up.

The way of producing nanostructured thin films described here allows sintering of the NP building blocks (in vertical and horizontal direction), achieved through inertial impaction during material production. The resulting films interact effectively with the target gas, attributing high sensitivity and fast-response sensing.^32,33^ The Pd NPs we produced here, ranging in size from 10 to 30 nm, can effectively adsorb hydrogen, enabling fast detection/sensing over a wide concentration range. The great advantage of our synthesis method is that it involves a single step, producing ready-to-use sintered NP-based films that can detect H_2_ at concentrations as low as 2 ppm. This is in the lower end of LoD values reported in the literature thus far (*i.e.*, from 0.4 to 600 ppm),^2,34–36^ making the proposed method highly promising.

In summary, the single-step synthesis method used in this work can successfully produce highly sintered NP-based thin films capable of detecting hydrogen at room temperature across a broad concentration range: *i.e.*, from approximately 2 ppm and up to a several percent. Considering the versatility and simplicity of the method to produce materials using NP building blocks of different composition and/or size, the performance of the sensors can in principle be further optimized and scaled up for industrial use.

## Data availability

The data supporting this article have been included as part of the ESI.†

## Conflicts of interest

There are no conflicts to declare.

## Supplementary Material

NA-OLF-D4NA00884G-s001
