# Supplementary material for: Single-step aerosol-based synthesis of nanostructured thin films for hydrogen sensing
Source: Nanoscale Adv. 2025 Feb 13;7(6):1505–8. doi: 10.1039/d4na00884g (PMC11831220; doi:10.1039/d4na00884g)
Supplement: NA-007-D4NA00884G-s001 [file NA-007-D4NA00884G-s001.pdf]

Supplementary Information (SI) for Nanoscale Advances.
This journal is © The Royal Society of Chemistry 2025

Supplementary information

Single-step Aerosol-based Synthesis of Nanostructured Thin Films for Hydrogen Sensing

Klito C. Petallidou,[a] Peter Kováčik,[b] Andreas Schmidt-Ott*[a,c,d] and George Biskos*[a,e]

[a]Climate and Atmosphere Research Centre, The Cyprus Institute, 2121 Nicosia, Cyprus,

[b]National Research Council of Canada, K1A 0R6, Ottawa, Canada,

[c]Faculty of Applied Sciences, Delft University of Technology, 2629 HZ Delft, The Netherlands,

[d]VSPARTICLE B.V, 2629 JD Delft, The Netherlands

[e]Faculty of Civil Engineering and Geosciences, Delft University of Technology, 2628 CN Delft, The Netherlands

Email: a.schmidt-ott@tudelft.nl (Andreas Schmidt-Ott); or

g.biskos@cyi.ac.cy; g.biskos@tudelft.nl (George Biskos)

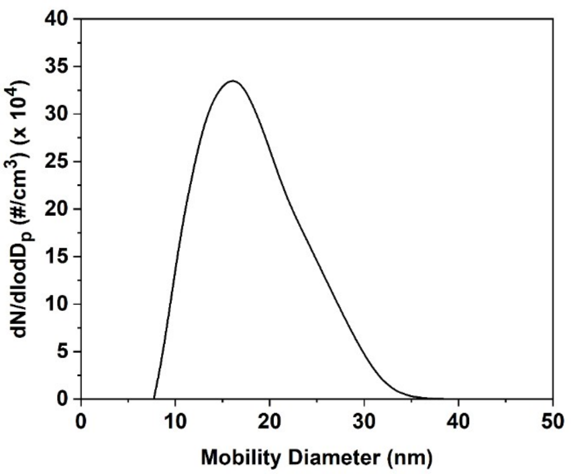

Figure S1. Size distribution of the Pd NPs produced by the glowing wire generator as those were measured by a Scanning Mobility Particle Sizer.
